# Case of Poliomyelitis Caused by Significantly Diverged Derivative of the Poliovirus Type 3 Vaccine Sabin Strain Circulating in the Orphanage

**DOI:** 10.3390/v12090970

**Published:** 2020-09-01

**Authors:** Ekaterina A. Korotkova, Maria A. Prostova, Anatoly P. Gmyl, Liubov I. Kozlovskaya, Tatiana P. Eremeeva, Olga Y. Baikova, Alexandr Y. Krasota, Nadezhda S. Morozova, Olga E. Ivanova

**Affiliations:** 1 Belozersky Institute of Physical-Chemical Biology, Lomonosov Moscow State University, 119899 Moscow, Russia; krasota@belozersky.msu.ru; 2 Federal State Budgetary Scientific Institution “Chumakov Federal Scientific Centre for Research and Development of Immune-and-Biological Products of the Russian Academy of Sciences” (FSBSI “Chumakov FSC R&D IBP RAS”), 108819 Moscow, Russia; prostovna@gmail.com (M.A.P.); lubov_i_k@mail.ru (L.I.K.); poliom_ldms@mail.ru (T.P.E.); baykovaaa@mail.ru (O.Y.B.); 3 Institute for Bionic Technologies and Engineering, Sechenov First Moscow State Medical University, 119991 Moscow, Russia; 4 Federal Centre of Hygiene and Epidemiology, Russian Federal Service for Surveillance on Consumer Rights Protection and Human Wellbeing, 117105 Moscow, Russia; morozova.n.s@mail.ru

**Keywords:** poliomyelitis, poliovirus, vaccine-derived poliovirus, VDPV, iVDPV, cVDPV, OPV, IPV, Sabin strains

## Abstract

Significantly divergent polioviruses (VDPV) derived from the oral poliovirus vaccine (OPV) from Sabin strains, like wild polioviruses, are capable of prolonged transmission and neuropathology. This is mainly shown for VDPV type 2. Here we describe a molecular-epidemiological investigation of a case of VDPV type 3 circulation leading to paralytic poliomyelitis in a child in an orphanage, where OPV has not been used. Samples of feces and blood serum from the patient and 52 contacts from the same orphanage were collected twice and investigated. The complete genome sequencing was performed for five polioviruses isolated from the patient and three contact children. The level of divergence of the genomes of the isolates corresponded to approximately 9–10 months of evolution. The presence of 61 common substitutions in all isolates indicated a common intermediate progenitor. The possibility of VDPV3 transmission from the excretor to susceptible recipients (unvaccinated against polio or vaccinated with inactivated poliovirus vaccine, IPV) with subsequent circulation in a closed children’s group was demonstrated. The study of the blood sera of orphanage residents at least twice vaccinated with IPV revealed the absence of neutralizing antibodies against at least two poliovirus serotypes in almost 20% of children. Therefore, a complete rejection of OPV vaccination can lead to a critical decrease in collective immunity level. The development of new poliovirus vaccines that create mucosal immunity for the adequate replacement of OPV from Sabin strains is necessary.

## 1. Introduction

The trivalent oral poliovirus vaccine (tOPV) from Sabin strains, which promotes the formation of both humoral and mucosal immunity in vaccine recipients, was considered the most effective tool for fulfilling the objectives of the Global Polio Eradication Initiative (GPEI) [1]. Due to its widespread use, the incidence of poliomyelitis caused by wild polioviruses was reduced 1000-fold [1], the eradication of wild polioviruses of types 2 [2] and 3 [3] was recognized, and four of the six WHO regions were certified as “polio-free” [4,5,6,7]. Against the background of these successes, the properties of tOPV as a vaccine derived from live attenuated strains turned out to be potentially dangerous. In very rare cases, it can lead to the development of vaccine-associated paralytic poliomyelitis (VAPP) in recipients or their nonvaccinated contacts [1]. In polio-free countries, including the Russian Federation [6], OPV remains the main source of poliovirus infection [8]. This situation cannot be corrected by the introduction of immunization schemes that use inactivated poliovirus vaccine (IPV) for primary vaccination [9], as the possibility of the infection of an unvaccinated contact remains. The ability of Sabin strains to evolve in the organism of the recipient or contacted persons leads to the formation of vaccine-derived polioviruses (VDPV) with restored neurovirulent properties [10]. Another source of VDPVs is people with primary immunodeficiency disorders (PID), in the organism of which Sabin strains can multiply for a long time, becoming a VDPV (iVDPV) [10]. Furthermore, a prerequisite for the formation of circulating VDPV (cVDPV) is the presence of a poorly vaccinated population. At the present time, these facts drive the epidemiological situation in the world, and have resulted in the return of poliomyelitis to many polio-free countries [11].

The threat of VDPV formation is most closely associated with the Sabin strain type 2 [10,12]. Thus, from 2000 to 2015, cVDPV outbreaks have occurred in 23 countries, and 87% of reported cases have been associated with type 2 [1]. After the replacement of tOPV in routine immunization programs with bivalent OPV from Sabin strains of types 1 and 3 [13], the situation only worsened: in 2016, cVDPVs type 2 were found in 2 countries, then in 2018 they were found in 7, then at the beginning of 2020 they were found in 15 [11]. The emergence of “new” cVDPVs type 2, which are the cause of outbreaks that occurred since 2016, is associated with immunization campaigns using mOPV2 to interrupt the circulation of type 2 cVDPV [14,15,16].

VDPVs originating from the Sabin strain type 3 are much less common. In the period 2000–2015, type 3 cVDPV outbreaks accounted for only 1.5% of known cVDPV cases [1]. Between 2016 and 2019, 7 acute flaccid paralysis (AFP) cases associated with cVDPV type 3 were recorded in one country (Somalia), compared with 522 cases associated with cVDPV type 2 in 18 countries [11]. As regards iVDPVs, since the year 1961, iVDPV type 2 has been the most prevalent (66% of the cases), followed by types 3 (17%), 1 (12%) and heterotypic mixes (i.e., types 1 and 2, or types 2 and 3–5%) [17]. The registration of cases with prolonged isolation of iVDPV by patients with PID is hard to account for completely due to the irregularity of registration of PID cases in different countries; nevertheless, it is estimated that from 1962 to 2018, 143 VDPV excretors with immunodeficiencies were identified in the world, 32 of which (22.4%) were long-term or chronic excretors of PV3 [18]. Only a few cases of high-level VDPV3 isolations from wastewater are known, the sources of which cannot be determined (the so-called aVDPV), but a high degree of divergence of which from Sabin strain type 3 suggests that their source was a person with immunodeficiency [19,20].

Although less than 1% of people with PID excrete iVDPV, the potential for iVDPV to enter the human population is considered a serious threat of OPV withdrawal after the global certification for polio eradication [18]. Currently, the ability of iVDPV to infect unvaccinated individuals in the surroundings of the excretor is rare [21]; it has been confirmed for poliovirus type 1 in a specific poorly vaccinated religious group [22] and poliovirus type 2 in a family [23]. In this report, we describe a poliovirus infection that spread in an orphanage, associated with VDPV type 3 which could have formed in an immunocompromised child.

## 2. Materials and Methods

### 2.1. The Acute Flaccid Paralysis Case

The AFP case (later defined as VAPP) was registered on 10 July 2014 in Syzran city hospital (Samara Region, Russia). The patient, a 13-months-old boy (P, patient), was born in a socially disadvantaged family. Since 7 May 2014 he lived in the Syzran orphanage that specializes in providing medical, pedagogical and social assistance to children aged up to 4 years with organic lesions of the central nervous system, mental disorders, and other congenital, hereditary and acquired diseases. The sanitary and hygienic conditions in the orphanage met the regulatory requirements of the Russian Federation. Children were separated into 4 isolated groups. Of the 54 children living in the orphanage, 26 (48%) were not vaccinated against poliomyelitis at the time of polio case occurrence. IPV was used in the orphanage for vaccination against poliomyelitis. However, 9 children (17%) had received from 1 to 3 doses of OPV (Table 1) before entering the orphanage.

The AFP case was detected and laboratory investigated according to the AFP assay algorithm adopted in Russia [24] and the WHO recommendations [25,26]. Feces samples: in total, 2 primary samples (11 July 2014 and 12 July 2014), samples collected on days 60, 90 and 120 after the paralysis onset, and three serum samples (14 July 2014; 24 July 2014 and 7 August 2014) were investigated. Healthy contacts of the patient were also examined, as follows: feces samples from 7 children living in the same room as the patient were collected on 12/13 July; stool and serum samples from all children in the orphanage (52 and 49, respectively) were investigated twice (3 and 4.5 months after the AFP case registration).

### 2.2. Virological Methods

The isolation and identification of viruses from fecal samples were performed by the standard procedures [26]. The viruses were isolated on RD, L20B and Hep-2c cell lines. Virus identification was performed in a neutralization assay [26]. The intratypic differentiation was carried out using a direct ELISA [26,27] and real-time RT-PCR [28].

### 2.3. Serological Methods

Poliovirus-neutralizing antibodies were determined in sera via neutralization assay with Sabin strains types 1, 2 and 3 in Hep-2c cells [25].

### 2.4. Genome Sequencing

The total RNA was isolated from the cultural medium of infected cells using the RNeasy minikit (Qiagen, Hilden, Germany), reverse–transcribed by the SuperScript reverse transcriptase (Invitrogen, Carlsbad, CA, USA) with random hexanucleotide primers (Syntol, Russia), amplified by PCR to produce 8 overlapping DNA fragments (primers are available upon request), and purified with QiaQuick DNA purification kit (Qiagen, Hilden, Germany). For sequencing, an ABI 3130 Genetic Analyzer was used.

### 2.5. Bioinformatics Methods

The full viral genomes were assembled using the Lasergene software (version 7.0.0; DNAStar, Madison, Wisconsin, USA). The sequence alignments and estimation of genomes divergence were performed with MEGA 6 [29].

### 2.6. GenBank Deposition

Nucleotide sequences were deposited in GenBank (accession numbers MT645947–MT645951).

## 3. Results

### 3.1. A Case of VAPP in a Small Cluster of VDPV Excretors

Flaccid lower paraparesis with lesions of the distal and proximal parts of the legs was detected in a child P (patient) living in the Syzran orphanage, 10 July 2014. Paresis arose sharply against the background of a fever within 7 days. Upon examination, the child showed a low tone of the lower extremities, a decrease in the knee reflexes and the absence of the Achilles reflexes. Sensitivity disorders and dysfunctions of the pelvic organs were not observed. Paresis of the extremities persisted on the 60th and 90th days after AFP onset. Before the disease, the boy was not vaccinated against polio. VDPVs type 3 was isolated from the fecal samples collected on 11 and 12 July. The AFP case was classified as “contact VAPP” (cVAPP) by the National Expert Classification committee as recommended by the WHO [30] and national regulations [31].

VDPVs type 3 were also found in fecal samples collected on 13 July from three out of seven investigated healthy contact infants from the same group in the orphanage. Two of these virus excretors (males C-1, 20 months old, and C-2, 23 months old) had previously been IPV-vaccinated, whereas the third one (female C-3, 20 months old) was not.

Two serological examinations of P carried out soon after the disease onset did not detect neutralizing antibodies against polioviruses of serotypes 1 and 2 (titers < 1:8), while the third test revealed a low titer of serotype 2 (1:16), and a significant accumulation of antibodies against the serotype 3 was recorded subsequently, from 1:32 (14 July) to 1:256 (24 July 7 August). Similarly, the serum of C-1, investigated on 7 August, contained antibodies only against serotype 3 (1:64) (Table 1), even though (as indicated in the documents) he was vaccinated with IPV in July and September, 2013. The simultaneously investigated serum of C-2 (who was also vaccinated twice with IPV in 2013) was positive for all three polio serotypes, with a somewhat higher titer to serotype 3 (Table 1). C-3 was unavailable at the time for the serological investigation.

### 3.2. Characterization of the Viral Isolates

The full genome sequences of the two isolates (separated by one day) from the VAPP patient and the isolates from three healthy contacts revealed that these viruses contained numerous mutations distinguishing them from the Sabin 3 strain (Table 2). All isolates contained 11 or more mutations in the capsid VP1 protein (Table S1), and were classified, according to the WHO-adopted criteria [32], as vaccine-derived polioviruses (VDPV) 

**Figure 1:**
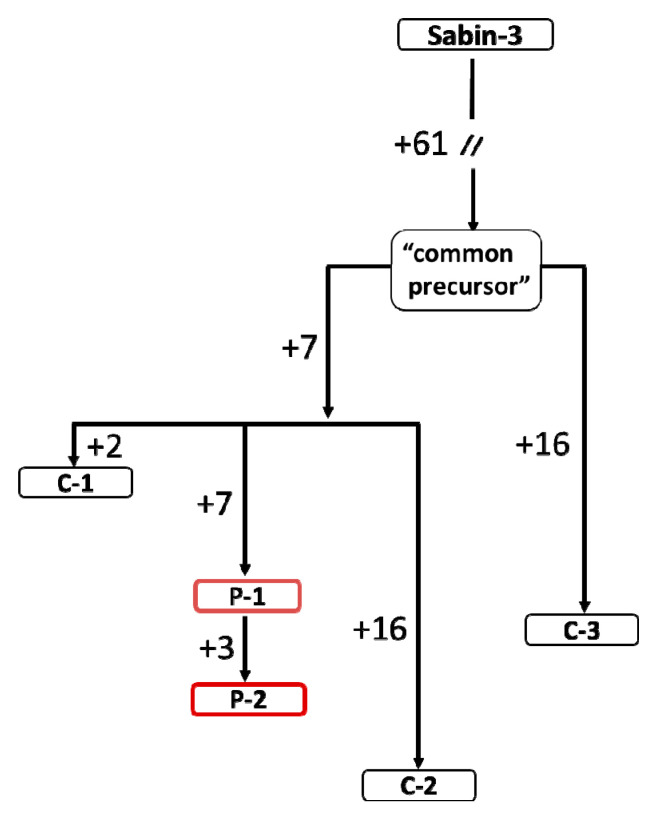
A hypothetical evolutionary pathway of the polioviruses isolated in Syzran in 2014. The identifiers of the isolates’ genomes are given in the frames. The numbers of the nucleotide mutations acquired by the isolates are indicated to the left of the arrows.

The minimal and maximal divergences of the genomes from those of the vaccine were 70 and 84 nt, in isolates from C-1 and C-2, respectively (Table 2). Although numerous mutations were excretor-specific, all the isolates shared 61 nucleotide substitutions, indicating that their divergent evolution from the OPV included a common intermediate predecessor (Table S1). The distribution of the mutations suggested that the isolates from C-1, P-1/P-2 and C-2 represented a lineage distinct from that of C-3, both descending from the intermediate (Figure 1). This former lineage appeared to undergo an additional short period of evolution, during which seven common mutations (none in VP1) had been accumulated. The most diverged viruses (C-2 and C-3), though isolated simultaneously, differed from one another at 39 nucleotide positions.

The relevant values markedly varied for the five isolates in the range of 8.8–10.6 months (Table 2). A larger part of this period (7.7 months) corresponded to the evolution from Sabin-3 to the common intermediate. Possibly, this is due to the different rates of divergence of the isolates via the “bottleneck” —the random selection of a part of the viral population when it passes from host to host, as well as by a different number of the intermediate hosts.

Some of the mutations were obviously biologically relevant, and at least a portion of them were of adaptive character. All isolates harbored deattenuating mutations in the 5′-UTR (U_472_C) and in the VP1-coding region (C_2493_U, resulting in Thr_6_Ile), both being commonly observed in the Sabin-3 derivates [34]. Moreover, the latter substitution is known to be already present in some batches of OPV [35]. The third adaptive mutation (U_2034_C, resulting in Phe_91_Ser substitution in VP3), which affects the temperature sensitivity of the poliovirus [36], was shared by all isolates but C-3 (Table S1).

Besides the above-mentioned adaptive mutations in the capsid proteins of all the isolates, there were substitutions in the antigenic sites. In the antigenic site 2 in VP2, there were Val_166_Ala substitutions (in the case of C-1, Asn_164_Ile as well), and we often observed in VDPVs the substitution of Ala_165_Thr in the adjoining antigenic site 2 position [37]. In the antigenic site 3 there were substitutions of Asn_288_Asp and Asp_290_Asn of VP1 and Ser_79_Leu in the antigenic site 4 of the VP3 of the isolate C-2. Moreover, all strains shared the Met_105_Thr substitution, often detected in VDPVs, [37] in the region of the VP1 protein involved in interactions with the poliovirus receptor [38].

In addition, several nucleotide mutations (G_162_A, G_395_A, U_472_C, U_480_C, U_5832_C and U_6938_C) are expected to stabilize the secondary RNA structure of domains II, IV and V of the 5′UTR [39], the RNase L competitive inhibitor RNA (ciRNA) *cis*-acting element [40], and the first conservative structural element of 3D-7000 [41] (Figure S1). All the isolates also have a mutation in 3A, which changes the Arg_15_Ser. It is believed that this substitution is the reason for the recombination of the derivatives of a vaccine strain of the type 3 [42].

### 3.3. Epidemiological Measures Carried Out in the Orphanage

The complex of anti-epidemic measures was carried out in the orphanage after the AFP case’s detection. It included the identification of healthy contacts (52 children) and the observation of their health, the laboratory investigation of specimens from the contacts (serum and feces samples), and the revision of the vaccination withdrawals.

In the summer–autumn of 2014, after the detection of the disease, 10, 23 and 7 children were given one, two or three doses of IPV, respectively. The first 11 children were vaccinated soon after the AFP case detection (14.07). At the end of July and in August, 8 children received IPV; in September, 21 did, in October 13 did and in November 24 did.

Fecal samples from 52 children were collected in October and additionally from 49 of them in November, and they were found to be poliovirus-negative.

The sera of all children were investigated for neutralizing antibody presence on 7–8 October and 24 November (Table 1). Several results deserve special attention. Judging by the documentation available, more than a half (30) of the 54 children were not vaccinated against polio before June 2014. Thus, this orphanage represented a rather effective domain for poliovirus transmission.

In October, there were four children testing negative for antibodies against serotypes 1 and 2, but exhibiting relatively high titers against serotype 3 (C-1, C-34, C-40, C-44), providing additional evidence that the transmission of VDPV-3 affected not only the poliovirus excretors detected. This notion is also in line with the fact that several sera (C-12, C-13, C-17, C-31, C-33) contained at least four-fold higher antibodies titers against serotype 3, compared to the other two.

## 4. Discussion

At the final stage of the Global Polio Eradication Initiative, when the victory over wild polioviruses of types 2 [2] and 3 [3] was announced, and wild poliovirus of type 1 circulated only in two countries (Afghanistan and Pakistan [43]), the problem of the derivatives of Sabin vaccine strains came to the fore. Long-evolved derivatives of Sabin strains, VDPVs, have the largest epidemiological significance [10,44]. The VDPVs include derivatives of the Sabin type 1 and Sabin type 3 strains that have accumulated at least 1% nucleotide substitutions in the region encoding the structural protein VP1, and variants of the Sabin type 2 strain with at least 0.6% mutations in the same genome region [32]. In 2018/2019, most poliomyelitis cases were not associated with wild polioviruses (33/173 cases, respectively), but with VDPVs instead (104/318 cases, respectively) [11,43]. Much less frequently, cases of poliomyelitis are associated with slightly altered variants of vaccine strains: as a rule, they cause sporadic cases of VAPP [45], but can also cause outbreaks [46].

Presented in this study, the case of a VDPV type 3 circulation in a specialized orphanage, which resulted in one child with VAPP, allows us to dwell on several points.

### 4.1. Genetic Features of Isolated Viruses

During their long evolution, the studied variants of the Sabin 3 strain accumulated a number of substitutions, some of which could cause the reversion of neurovirulence and the possibility of prolonged transmission. In addition to the known adaptive mutations (already sufficient to significantly increase the viability of the virus [47]), all isolates have a large number of nonsynonymous substitutions in the antigenic sites, which could provide the ability to escape the immune response. In addition, a number of mutations were found in the isolates that strengthen the secondary structure of RNA in 5′-UTR, in the *cis* element-inhibiting RNaseL activity located in the 3C region and in the first conservative structural element of 3D-7000. Many of these mutations were also observed in other variants of Sabin 3 (Table S2).

Another interesting feature of the studied isolates (non-recombinants) is an Arg_15_Ser mutation in protein 3A. Most (about 80%) of the tested type 3 natural isolates of vaccine origin are intertype recombinants [48,49]. Earlier, it was suggested that by changing certain parts of the genome to the sequences of other types, recombinant viral variants obtained an advantage over non-recombinants [42]. The genome region, which is usually replaced in the studied type 3 recombinants, contains the only significant difference of the Sabin-3 strain and its wild predecessor from other polioviruses: the 15th arginine in 3A. The assumption was made that, by getting rid of precisely this difference, recombinant vaccine variants of type 3 acquire a selective advantage. Previously, it was not possible to detect a point mutation that changes the 15th arginine in 3A in non-recombinant natural type 3 isolates. However, all isolates considered here have such a substitution, which supports our earlier assumption of its adaptive value for vaccine-derived polioviruses of type 3. In addition, this finding shows that viruses can improve their viability by replacing the failed site through both recombination and mutational variability.

### 4.2. Epidemiological Assessment of the Situation

We believe that the origin of the VDPV circulation observed in the orphanage could be Sabin-3, which evolved over a long period in the organism, most likely, of one host. In favor of the assumption that the “common ancestor” of the isolates is iVDPV is the fact that all isolates are not recombinants, and, moreover, we have not found any other related viruses. The fact that the virus was actively circulating in the orphanage (cVDPV) is confirmed by both the isolation of related but significantly different viruses from four children (Table S1), and by a higher level of antibodies against poliovirus type 3 compared to other serotypes in a significant number of the children living in the institution (Table 1). Thus, we have a reason to conclude that we have observed the transition of iVDPV to cVDPV.

Unfortunately, we were not able to identify the source of the infection, as only IPV was used for vaccination in the orphanage. It is feasible that one of the newly enrolled children acquired the virus through contact in a previous place of residence, or in a medical facility. Long-term poliovirus excretion, exceeding the quarantine time upon admission (one month), is not a rare event. This is especially true for weakened children with various severe diagnoses, for whom this specialized orphanage is intended. It should be noted that at the time of the disease detection, nine children previously routinely vaccinated with OPV lived in the orphanage. Genetic analysis of the isolates showed that the time of their divergence from the vaccine strain ranges from 9 to 11 months (Table 2). In four of the nine children previously vaccinated with a live poliovirus vaccine, the period from one of the OPV vaccinations to the case registration (8, 9, 9 and 11 months) corresponds to the calculated “age” of the isolated polioviruses. It cannot be ruled out that one of these four OPV recipients could be the source of infection, primarily C-6, who lived in the same group as the patient and with three other children excreting the poliovirus.

The case presented here in accordance with current WHO recommendations [14] can be classified as an outbreak of poliovirus infection caused by iVDPV type 3, which in a closed childcare institution has been able to circulate among both unvaccinated and vaccinated children, and therefore, should be considered as cVDPV type 3.

### 4.3. Implications for the Development of Vaccination Strategy for the Polio Eradication Program

A frightening discovery in the investigation of this epidemiological situation was the lack of immunity to at least two poliovirus serotypes in a significant number of children at least twice vaccinated with IPV when the assessment of antibody levels was carried out more than a month after the first vaccination (10 people, almost 20% (Table 1)). Moreover, four doses of inactivated vaccine in C-1 and two doses of IPV, plus three doses of OPV in C-5, failed to produce immunity to polioviruses. The investigation of the level of immunoglobulins A, G and M excluded humoral immunodeficiency in these children. It can be assumed that severe somatic pathology in young children makes it difficult for them to generate the specific response to the vaccine.

According to the WHO’s current plans, after the global OPV cessation, polio immunization will only be carried out with IPV [50], and at least two doses of trivalent IPV are recommended for immunization schedules [51]. However, our observation shows that two IPV doses may not be enough to form immunity to polioviruses (at least in special contingents of children), which can be a significant danger in the case of the contact of non-immune children with an infectious agent. No less important is the economic aspect of such a transition: it must be assumed that the vaccination schedule using IPV cannot be limited to only two doses, and this will lead to a rise in the cost of vaccination and will require an increase in the scale of IPV production. Schemes of vaccination against polio in developed countries with good vaccination coverage now include from four to six doses of IPV, while the introduction of the one IPV dose into the vaccination schedules of most countries, including the most vulnerable countries in Asia and Africa, carried on for 3 years after the switch from trivalent OPV to bivalent OPV. All the time, there was a significant deficit in IPV. Can we be sure that the capabilities of existing IPV manufacturers will be able to meet the increasing need for the vaccine?

It has been convincingly shown that the introduction of polioviruses into the non-immune group leads to its rapid spread and prolonged circulation [52]. At the time of the planned global transition from OPV to the exclusive use of IPV, the world’s population will have a different vaccination status: those who received OPV, vaccinated only with IPV, and not vaccinated at all (and the number of such children is increasing every year). Furthermore, a significant proportion of people will have no intestinal immunity. At the same time, a potential source of poliovirus infection (vaccine-derived viruses circulating in certain regions, patients diagnosed with immunodeficiency, research laboratories and vaccine manufacturers) will exist for a long time. The result of the hasty rejection of OPV may lead to new polio outbreaks, the fight against which will have to start almost from scratch. Consequently, urgent completion of work on a new and improved vaccine is needed, aimed at creating not only humoral, but also mucosal immunity, on the one hand, that is safer than Sabin strains on the other hand. Investigations in this direction are being actively carried out by several research groups [53,54,55,56]. Switching exclusively to IPV, in a situation wherein the possibility of introducing any type of Sabin-related viruses and their further spread and transformation into VDPVs is not completely ruled out, can lead to a disaster.

### 4.4. Importance for Surveillance of Poliovirus Circulation

The main type of surveillance of poliovirus circulation for implementing the Global Polio Eradication Initiative is the surveillance of acute flaccid paralysis (AFP). However, over time, with the further implementation of the Program, additional types of surveillance, aimed at identifying the long-term asymptomatic circulation of polioviruses, has become increasingly important. This includes environmental surveillance (wastewater research) and the study of PID patients as potential poliovirus excretors [50]. Russia participated in two WHO collaborative studies to search for such patients, but no long-term or chronic poliovirus excretors were found [57,58]. The identification of patients with PID is still a rather difficult task in many countries, since it largely depends on the level of development of the country and the level of health care. However, in recent years, the registration of PID patients in middle-income countries has increased [59]. These countries include Russia, which continues to use OPV. Thus, one would expect that in Russia there may be patients continuously or chronically excreting polioviruses. The validity of this assumption is evidenced by the fact that the significantly divergent poliovirus type 2 (17.6% nt substitutions in VP1) was detected in Moscow wastewater [60]. Our current observation confirms the danger of the prolonged excretion of poliovirus by persons with immunodeficiency conditions, and the need for surveillance for iVDPV.

## Figures and Tables

**Table 1:** Number of doses of inactivated poliovirus vaccine (IPV) and oral poliovirus vaccine (OPV) and level of neutralizing antibodies against polioviruses types 1, 2 and 3 in the sera of the contacts (arranged in accordance of the poliovaccine doses acquired).

The Contacts (Age in Months at the Time of the Acute Flaccid Paralysis (AFP) Case Detection)	Number of IPV (+OPV) Doses before the First Serological Examination	Antibody Titer (7–8 October 2014) against Poliovirus Serotype:			Number of IPV Doses between Two Serological Examinations	Antibody Titer (24 November 2014) against Poliovirus Serotype:		
		1	2	3		1	2	3
C-4 (23)	2 (+3)	1:64	1:256	1:32	0	ND ***	ND	ND
C-5 (41)	2 (+3)	<1:8	1:128	<1:8	0	<1:8	1:256	<1:8
C-6 * (31)	2 (+3)	1:512	1:512	1:1024	0	1:256	1:256	1:512
C-7 (31)	3 (+2)	1:512	1:512	1:512	0	1:512	1:256	1:256
C-8 (33)	3 (+2)	1:32	1:128	1:256	0	1:64	1:128	1:128
C-9 (41)	3 (+2)	1:64	1:64	1:16	0	1:64	1:64	1:8
C-10 (36)	4 (+1)	1:512	1:512	1:128	0	1:256	1:512	1:256
C-11 (12)	2 (+1)	1:32	1:64	1:16	0	1:16	1:64	1:16
C-12 (13)	2 (+1)	1:64	1:64	1:512	1	1:512	1:512	1:512
C-13 (30)	6	1:64	1:256	>1:1024	0	1:64	1:128	1:256
C-14 (25)	6	1:128	1:128	1:128	0	1:64	1:128	1:64
C-15 (44)	5	>1:1024	1:512	1:512	0	>1:1024	1:512	1:512
C-16 * (25)	5	1:512	1:512	1:256	0	1:128	1:256	1:128
C-17 (31)	5	1:64	1:32	1:512	0	1:32	1:64	1:256
C-18 (41)	5	1:64	1:128	1:256	0	1:64	1:256	1:128
C-19 (47)	5	1:32	1:256	1:64	0	1:64	1:256	1:64
C-20 (20)	5	1:64	1:128	1:64	0	1:64	1:128	1:64
C-21 (33)	5	1:32	1:128	1:16	0	1:16	1:64	<1:8
C-22 (24)	4	>1:1024	1:1024	1:256	0	1:512	1:256	1:128
C-23 (21)	4	1:64	1:128	1:256	0	1:256	1:64	1:256
C-2 ** (22)	4	1:64	1:128	1:256	0	1:16	1:16	1:128
C-1 ** (20)	4	<1:8	<1:8	1:64	1	1:64	1:16	1:64
C-24 (21)	3	1:1024	1:256	1:512	0	1:256	1:256	1:128
C-25 (11)	3	1:256	1:512	1:256	0	1:32	1:128	1:64
C-26 * (23)	3	1:256	1:256	1:512	0	1:128	1:256	1:128
C-27 (52)	2	1:512	1:1024	1:512	1	1:128	1:256	1:512
C-28 (9)	2	1:256	1:256	1:128	1	1:256	1:256	1:64
C-29 (10)	2	1:64	1:256	1:128	1	1:64	1:256	1:512
C-30 (43)	2	1:32	1:256	1:256	1	1:16	1:128	1:512
C-31 (11)	2	1:16	1:32	1:256	1	1:128	1:128	1:256
C-32 (9)	2	1:32	1:128	1:64	1	1:256	1:512	1:256
C-33 (9)	2	<1:8	1:16	1:256	1	1:8	1:32	1:256
C-34 (14)	2	<1:8	<1:8	1:128	1	1:64	1:32	1:256
C-35 * (5)	1	1:512	1:512	1:128	1	1:256	1:256	1:16
C-36 (1)	1	1:64	1:128	1:64	1	1:512	1:512	1:256
C-37 (24)	1	1:32	1:16	1:8	1	1:128	1:128	1:32
C-38 (3)	1	1:8	1:64	1:32	1	1:256	1:256	1:256
C-39 (38)	1	1:8	1:64	1:16	1	1:128	1:64	1:8
C-40 (13)	1	<1:8	1:8	1:128	1	1:64	1:64	1:512
C-41 (1)	1	<1:8	1:32	<1:8	1	1:128	1:64	1:128
C-42 (4)	1	<1:8	1:8	<1:8	2	1:64	1:128	1:32
C-43 (4)	1	<1:8	<1:8	<1:8	1	1:64	1:64	1:64
C-44 (23)	1	<1:8	1:8	1:256	0	ND	ND	ND
C-45 (1)	1	<1:8	<1:8	<1:8	0	ND	ND	ND
C-46 (0)	0	<1:8	<1:8	<1:8	2	1:16	1:16	<1:8
C-47 (35)	0	<1:8	<1:8	<1:8	2	1:8	<1:8	1:8
C-48 (13)	0	<1:8	<1:8	<1:8	2	<1:8	<1:8	1:8
C-49 (2)	0	<1:8	<1:8	<1:8	2	<1:8	<1:8	<1:8
C-50 (6)	0	<1:8	<1:8	<1:8	2	<1:8	<1:8	<1:8
C-51 (7)	0	<1:8	<1:8	<1:8	2	<1:8	<1:8	<1:8
C-52 (8)	0	<1:8	<1:8	<1:8	2	<1:8	<1:8	<1:8
C-53 (16)	0	<1:8	<1:8	<1:8	2	<1:8	<1:8	<1:8

* Children living in the same room with the patient; ** Poliovirus excretors; *** ND—not done, sera were not collected and investigated.

**Table 2:** Ages of Syzran isolates and their hypothetical common precursor.

Isolate Genome	Number of Mutations Per Genome	Approximated Age, Months *
“Common ancestor”	61	7.7
P-1	75	9.5
P-2	78	9.8
C-1	70	8.8
C-2	84	10.6
C-3	77	9.7

* The age was estimated according to Sabin-3 by the approximate rate of 1.28·10^2^ substitutions per site per year [33].

## Supplementary Materials

The following are available online at https://www.mdpi.com/1999-4915/12/9/970/s1, Figure S1: Nucleotide substitutions in the phylogenetically conserved RNA structural elements of the genomes of the poliovirus isolates, in comparison with the Sabin-3 strain, Table S1: Mutations in the genomes of the Syzran isolates, Table S2: Mutations in the genomes of Syzran isolates found in other vaccine-related polioviruses type 3.
