# Supplementary material for: Case of Poliomyelitis Caused by Significantly Diverged Derivative of the Poliovirus Type 3 Vaccine Sabin Strain Circulating in the Orphanage
Source: Viruses. 2020 Sep 1;12(9):970. doi: 10.3390/v12090970 (PMC7552002; doi:10.3390/v12090970)
Supplement: Supplementary file 1 [file viruses-12-00970-s001.pdf]

viruses MDPI

Case of poliomyelitis caused by significantly diverged derivative of the poliovirus type 3 vaccine Sabin strain circulating in the orphanage

Ekaterina A. Korotkova[1,*], Maria A. Prostova[2,a], Anatoly P. Gmyl[2,3,b], Liubov I. Kozlovskaya[2,3], Tatiana P. Eremeeva[2], Olga Y. Baikova[2], Alexandr Y. Krasota[1,2], Nadezhda S. Morozova[4], and Olga E. Ivanova[2,3,*]

1 Belozersky Institute of Physical-Chemical Biology, Lomonosov Moscow State University, Moscow, 119899, Russia; katerkorot@yandex.ru (E.A.K.)
2 Federal State Budgetary Scientific Institution "Chumakov Federal Scientific Centre for Research and Development of Immune-and-Biological Products of the Russian Academy of Sciences" (FSBSI "Chumakov FSC R&D IBP RAS"), Moscow, 108819, Russia; (A.P.G.); prostovna@gmail.com (M.A.P.); lubov_i_k@mail.ru (L.I.K.); poliom_ldms@mail.ru (T.P.E.); baykovaaa@mail.ru (O.Y.B.); krasota@belozersky.msu.ru (A.Y.K.); ivanova_oe@chumakovs.su (O.E.I.)
3 Sechenov First Moscow State Medical University, Moscow, 119991, Russia (O.E.I.; L.I.K.)
4 Federal Centre of Hygiene and Epidemiology, Russian Federal Service for Surveillance on Consumer Rights Protection and Human Wellbeing, Moscow,117105, Russia; morozova.n.s@mail.ru (N.S.M.)
a Now works at Institute of Molecular Genetics of the Russian Academy of Sciences, Moscow, 123182, Russia
b Deceased

* Correspondence: katerkorot@yandex.ru; Tel.: +7- 916-169-86-12 (E.A.K.)
ivanova_oe@chumakovs.su ; Tel.: +7-916-677-24-03 (O.E.I.)

Supplementary material

Table S1. Mutations in the genomes of the Syzran isolates

Genome region	Nucleotide (amino acid) mutation	Genome(s) of Syzran isolates harboring mutation
5′-UTR	$G_{113} \to A$	all
	$C_{123} \to U$	C-2
	$G_{162} \to A$	all but C-3
	$G_{189} \to C$	P-2
	$C_{346} \to U$	C-2
	$G_{395} \to A$	all
	$U_{472} \to C$*	all
	$U_{480} \to C$	all
	$U_{602} \to C$	all
	$G_{673} \to G/A$	C-3
	$U_{708} \to C$	C-3
	$U_{738} \to C$	C-3
VP4	$A_{772} \to G$	all
VP2	$A_{964} \to G$	C-3
	$C_{1035} \to U$ ($A_{29} \to V$)	P-1 and P-2
	$C_{1084} \to U$	C-2
	$U_{1126} \to C$	C-3
	$C_{1141} \to U$	C-3
	$A_{1252} \to G$	all
	$G_{1390} \to A$	all
	$A_{1440} \to U$ ($N_{164} \to I$)**	C-1
	$G_{1442} \to A$ ($A_{165} \to T$)**	all
	$U_{1446} \to C$ ($V_{166} \to A$)**	all
	$G_{1477} \to A$	all
	$U_{1540} \to C$	all
	$U_{1543} \to C$	all
	$U_{1591} \to C$	P-1 and P-2
	$U_{1592} \to C$ ($L_{215} \to M$)	all
	$C_{1596} \to U$ ($A_{216} \to V$)	C-3
VP3	$C_{1795} \to U$	all but C-3
	$G_{1807} \to A$	C-2
	$U_{1855} \to C$	C-2
	$C_{1998} \to U$ ($S_{79} \to L$)**	C-2
	$U_{2034} \to C$ ($F_{91} \to S$)*	all but C-3
	$U_{2299} \to C$	all but C-3
	$U_{2340} \to A$ ($F_{193} \to Y$)	C-3
	$U_{2413} \to C$	all
VP1	$C_{2493} \to U$ ($T_{6} \to I$)*	all
	$U_{2569} \to C$	P-2
	$A_{2599} \to G$	all
	$C_{2611} \to U$	all
	$C_{2637} \to U$ ($A_{54} \to V$)	all
	$A_{2638} \to G$	C-2
	$U_{2693} \to A$ ($S_{73} \to T$)	all
	$A_{2762} \to G$ ($T_{96} \to A$)	all
	$U_{2790} \to C$ ($M_{105} \to T$)	all
	$G_{2959} \to A$	all
	$G_{2974} \to A$	all
	$A_{3338} \to G$ ($N_{288} \to D$)**	all
	$G_{3344} \to A$ ($D_{290} \to N$)**	all
	$U_{3376} \to C$	P-1 and P-2
2A	$C_{3522} \to U$ ($T_{49} \to I$)	all
	$G_{3579} \to A$ ($R_{68} \to K$)	C-2
	$G_{3676} \to U$	all
	$U_{3715} \to C$	C-2
	$U_{3778} \to C$	all but C-3
2B	$U_{3838} \to C$	all
	$G_{4066} \to A$	all
2C	$G_{4342} \to A$	all
	$A_{4417} \to C$	all
	$A_{4433} \to G$ ($T_{107} \to A$)	all
	$A_{4612} \to U$	C-3
	$G_{4630} \to A$	all
	$U_{4663} \to C$	all
	$A_{4771} \to G$	P-1 and P-2
	$C_{4774} \to U$	C-2
	$C_{4786} \to U$	P-2
	$U_{4849} \to C$	all
	$A_{4861} \to G$	all
	$G_{4868} \to A$ ($G_{252} \to S$)	all
	$A_{4872} \to G$ ($E_{253} \to G$)	all
	$C_{4917} \to U$ ($T_{268} \to M$)	all
	$U_{5035} \to C$	P-1 and P-2

*Adaptive mutations

**Mutations in the antigenic sites

Genome region	Nucleotide (amino acid) mutation	Genome(s) of Syzran isolates harboring mutation
3A	$C_{5116}\rightarrow U$	C-3
	$A_{5119}\rightarrow G$	P-1 and P-2
	$G_{5146}\rightarrow U$ ($R_{15}\rightarrow S$)	all
	$A_{5158}\rightarrow G$	P-1 and P-2
	$C_{5357}\rightarrow U$ ($H_{86}\rightarrow Y$)	C-3
3B	$A_{5365}\rightarrow G$	C-2
	$C_{5386}\rightarrow U$	all
	$U_{5398}\rightarrow C$	all
	$G_{5425}\rightarrow A$	all
3C	$C_{5524}\rightarrow U$	all
	$A_{5567}\rightarrow G$ ($I_{47}\rightarrow V$)	all
	$C_{5597}\rightarrow U$	C-2
	$C_{5602}\rightarrow A$ ($D_{58}\rightarrow E$)	all but C-3
	$C_{5677}\rightarrow U$	all
	$U_{5832}\rightarrow C$ ($I_{135}\rightarrow T$)	all
	$C_{5854}\rightarrow U$	all

Genome region	Nucleotide (amino acid) mutation	Genome(s) of Syzran isolates harboring mutation
3D	$G_{6086}\rightarrow A$ ($V_{37}\rightarrow I$)	all
	$A_{6109}\rightarrow G$	all
	$U_{6157}\rightarrow C$	all but C-3
	$U_{6244}\rightarrow C$	all
	$C_{6266}\rightarrow C/U$	C-3
	$G_{6304}\rightarrow C$	all
	$C_{6337}\rightarrow U$	C-2
	$U_{6364}\rightarrow U/C$	C-3
	$A_{6490}\rightarrow G$	all
	$U_{6550}\rightarrow C$	all
	$U_{6562}\rightarrow C$	all
	$C_{6589}\rightarrow U$	all
	$C_{6592}\rightarrow U$	C-2
	$A_{6658}\rightarrow G$	C-2
	$A_{6664}\rightarrow A/G$	C-3
	$C_{6769}\rightarrow U$	all
	$U_{6877}\rightarrow C$	all
	$A_{6918}\rightarrow A/G$ ($K_{314}\rightarrow K/R$)	C-3
	$U_{6938}\rightarrow C$	all
	$U_{7291}\rightarrow C$	all
	$A_{7303}\rightarrow A/G$	C-3
	$A_{7321}\rightarrow A/U$	C-1
	$U_{7360}\rightarrow C$	C-2

*Adaptive mutations
**Mutations in the antigenic sites

Table S2. Mutations* in the genomes of Syzran isolates found in other vaccine-related polioviruses type 3.

Genome region	Nucleotide (amino acid) mutation	Genome(s) of Syzran isolates harboring mutation	Known or assumed biological relevance of mutation	Previous report (s) of same mutation in Sabin-3-derived viruses
5′-UTR	G_{162}→A	all but C-3	Stabilization of domain II structure	1, 2
	G_{395}→A	all	Stabilization of domain IV structure	1-4
	U_{480}→C	all	Stabilization of domain V structure	1, 2
	U_{602}→C	all	–	1, 5
	U_{738}→C	C-3	–	1
VP4	A_{772}→G	all	–	4
VP2	A_{964}→G	C-3	–	2
	C_{1035}→U (A_{29}→V)	P-1, P-2	–	6
	G_{1390}→A	all	–	2
	G_{1442}→A (A_{165}→T)	all	Region of antigenic site 2	1, 7**, 8
	U_{1446}→C (V_{166}→A)	all	Antigenic site 2	1, 7
	U_{1592}→C (L_{215}→M)	all	–	1
VP3	C_{1795}→U	all but C-3	–	1
	C_{1998}→U (S_{79}→L)	C-2	Antigenic site 4	9
	U_{2299}→C	all but C-3	–	1, 2
	U_{2413}→C	all	–	5
VP1	C_{2637}→U (A_{54}→V)	all	–	1, 2, 4, 5, 9, 10
	U_{2790}→C (M_{105}→T)	all	Interaction with poliovirus receptor	1, 2, 3, 5, 7, 9,10
	A_{3338}→G (N_{288}→D)	all	Antigenic site 3	1, 2, 9
	G_{3344}→A (D_{290}→N)	all	Antigenic site 3	1
2A	G_{3676}→U	all	–	1
	U_{3778}→C	all but C-3	–	2
2C	A_{4433}→G (T_{107}→A)	all	–	1
	C_{4917}→U (T_{268}→M)	all	–	4, 9
3C	U_{5832}→C (I_{135}→T)	all	Stabilization of ciRNA structure	1, 11

*Without three known adaptive mutations observed in most variants of Sabin-3.

**In this article only amino acid substitutions were listed.

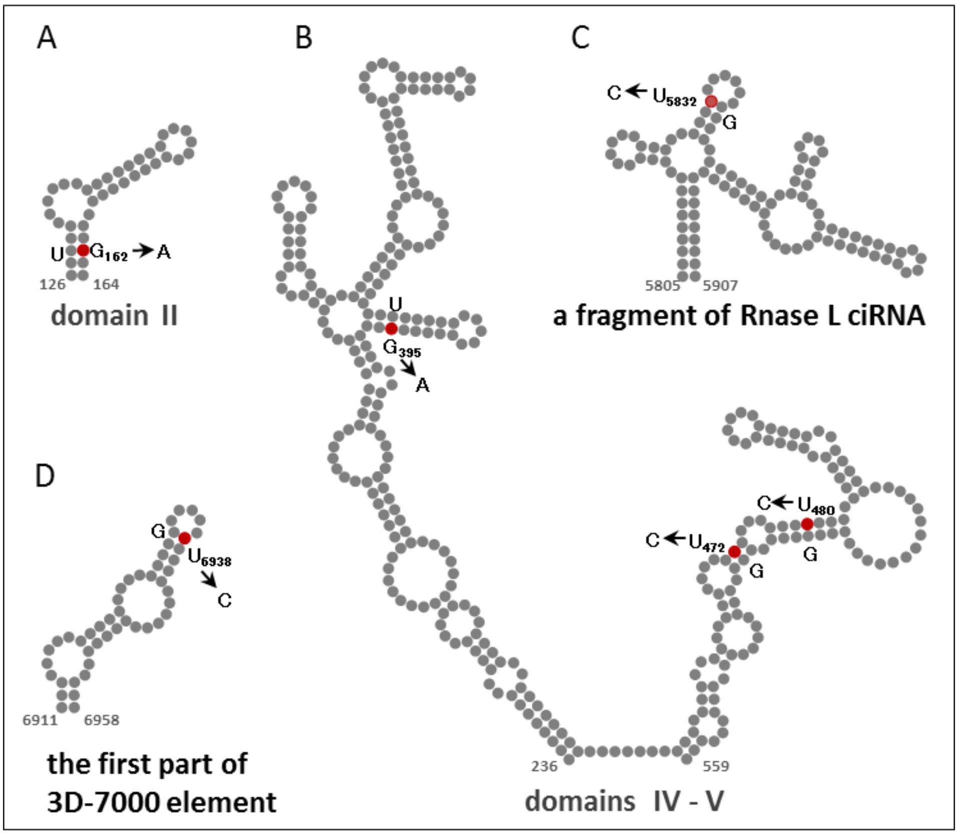

Figure S1. Nucleotide substitutions in the phylogenetically conserved RNA structural elements of the genomes of the poliovirus isolates, in comparison with the Sabin-3 strain. The positions of changed nucleotides are marked in red, and corresponding mutations are indicated; the nucleotides involved in the resulting base-pairing are also shown. A. Domain II of 5′UTR. B. Domains IV-V of 5′UTR [12]. C. A fragment of Rnase L competitive inhibitor element of viral RNA (ciRNA) [13]. D. The first conservative structural element of 3D-7000 [14].

References

1. Laassri M.; Zagorodnyaya T.; Chumakov K. Vaccine Derived Poliovirus RNA Sequences (Unpublished).
2. Rakoto-Andrianarivelo M.; Gumede N.; Jegouic S.; Balanant J.; Andriamamonjy S.N.; Rabemanantsoa S.; Birmingham M.; Randriamanalina B.; Nkolomoni L.; Venter M.; Schoub B.D.; Delpeyroux F.; Reynes J.M. Reemergence of recombinant vaccine-derived poliovirus outbreak in Madagascar. *J Infect Dis*. **2008**, 197, 1427-1435, doi: 10.1086/587694.
3. Blomqvist S.; Savolainen-Kopra C.; Paananen A.; El Bassioni L.; El Maamoon Nasr E.M.; Firstova L.; Zamiatina N.; Kutateladze T.; Roivainen M. Recurrent isolation of poliovirus 3 strains with chimeric capsid protein Vp1 suggests a re-combination hot-spot site in Vp1. *Virus Res*. **2010**, 151, 246–251, doi: 10.1016/j.virusres.2010.05.006.
4. Shahmahmoodi S.; Parvaneh N.; Burns C.; Asghar H.; Mamishi S.;Tabatabaie H.; Chen Q.; Teimourian S.; Gooya M.M.; Esteghamati A.R.; Mousavi T.; Yousefi M.; Farrokhi K.; Mashlool M.; Kew O.; Nategh R. Isolation of a type 3 vaccine-derived poliovirus (VDPV) from an Iranian child with X-linked agammaglobulinemia. *Virus Res*. **2008**, *137*, 168-172, doi: 10.1016/j.virusres.2008.07.006.
5. Savolainen-Kopra C.; Samoilovich E.; Kahelin H.; Hiekka A.K.; Hovi T.; Roivainen M. Comparison of poliovirus recombinants: accumulation of point mutations provides further advantages. *J Gen Virol*. **2009**, 90, 1859-1868, doi: 10.1099/vir.0.010942-0.
6. Cassemiro K.M.S.M.; Burlandy F.M.; da Silva EE. Rare natural type 3/type 2 intertypic capsid recombinant vaccine-related poliovirus isolated from a case of acute flaccid paralysis in Brazil, 2015. *J Gen Virol*. **2016**, 97, 1545-1550, doi: 10.1099/jgv.0.000484.
7. Yakovenko M.L.; Cherkasova E.A.; Rezapkin G.V.; Ivanova O.E.; Ivanov A.P.; Eremeeva T.P.; Baykova O.Y.; Chumakov K.M.; Agol V.I. Antigenic evolution of vaccine-derived polioviruses: changes in individual epitopes and relative stability of the overall immunological properties. *J Virol*. **2006**, *80*, 2641–2653, doi:10.1128/JVI.80.6.2641-2653.2006.
8. Blomqvist S.; Bruu A.L.; Stenvik M.; Hovi T. Characterization of a recombinant type 3/type 2 poliovirus isolated from a healthy vaccinee and containing a chimeric capsid protein VP1. *J Gen Virol*. **2003**, 84, 573-580, doi: 10.1099/vir.0.18708-0.
9. World Health Organization (WHO). Report of the second meeting of the technical consultation group for global eradication of poliomyelitis. WHO/EPI/GEN/98/04. Geneva, Switzerland, 1998. Available online: http://apps.who.int/iris/bitstream/10665/63994/1/WHO_EPI_GEN_98.04.pdf])
10. Cherkasova E.; Laassri M.; Chizhikov V.; Korotkova E.; Dragunsky E.; Agol V.I.; Chumakov K. Microarray analysis of evolution of RNA viruses: evidence of circulation of virulent highly divergent vaccine-derived polioviruses. *Proc Natl Acad Sci USA*. **2003**, 100, 9398-9403, doi: 10.1073/pnas.1633511100.
11. Famulare M.; Chang S.; Iber J.; Zhao K.; Adeniji J.A.; Bukbuk D.; Baba M.; Behrend M.; Burns C.C.; Oberste M.S. Sabin vaccine reversion in the field: a comprehensive analysis of Sabin-like poliovirus isolates in Nigeria. *J Virol*, **2015**, 90, 317-331, doi: 10.1128/JVI.01532-15.
12. Pilipenko E.V.; Blinov V.M.; Romanova L.I.; Sinyakov A.N.; Maslova S.V. Agol V.I. Conserved structural domains in the 5′-untranslated region of picornaviral genomes: an analysis of the segment controlling translation and neurovirulence. *Virology*. **1989**, *168*, 201–209, doi:10.1016/0042-6822(89)90259-6.
13. Han J.Q.; Townsend H.L.; Jha B.K.; Paranjape J.M.; Silverman R.H.; Barton D.J. A phylogenetically conserved RNA structure in the poliovirus open reading frame inhibits the antiviral endoribonuclease RNase L. *J Virol*. **2007**, *81*, 5561–5572, doi:10.1128/JVI.01857-06.
14. Burrill C.P.; Westesson O.; Schulte M.B.; Strings V.R.; Segal M.; Andino R.J. Global RNA structure analysis of poliovirus identifies a conserved RNA structure involved in viral replication and infectivity. *Virology*. **2013**, *87*, 11670-11683, doi: 10.1128/JVI.01560-13.
